# Retrospective study on pain management pathway for patients with suspected renal/ ureteric colic in a U.K. accidents and emergency department: A quality improvement project

**DOI:** 10.12688/f1000research.152325.1

**Published:** 2024-09-12

**Authors:** Charles Ojo, Chijioke Orji, Olorungbami Anifalaje, Gourav Garg, Mariette Anto, Megan Kelly

**Affiliations:** 1 Emergency Department, Pilgrim Hospital, United Lincolnshire Hospitals NHS Trust, Boston, Lincolnshire, PE21 9QS, UK; 2 Trauma and Orthopaedics, Royal Cornwall Hospital, Truro, England, TR1 3LJ, UK; 3 Emergency Department, Dumfies and Galloway Royal Infirmary, Cargenbridge, Dumfries, DG2 8RX, UK; 4 Emergency Department, King's Mill Hospital, Sherwood Forest Hospitals NHS Foundation Trust, Sutton-in-Ashfield, Nottinghamshire, NG17 4JL, UK

**Keywords:** Renal, ureteric, colic, stones, NSAIDs, paracetamol, opioids, CT-KUB.

## Abstract

**Background:**

In acute settings around the globe, renal/ureteric colic remains one of the most common diagnoses for patients presenting with loin-to-groin pain. Even though management spans from medical expulsive therapies to surgical options, pain as a significant component of a patient’s presentation must be dealt with quickly, decisively, and safely, as it can be excruciating and its effects on overall health are dire. This study aimed to explore compliance with the National Institute for Health and Care Excellence (NICE) guidelines for pain management in patients with suspected renal/ureteric colic. It includes the use of nonsteroidal anti-inflammatory drugs (NSAIDs), intravenous paracetamol, and opioids as first-, second-, and third-line analgesics, respectively, and does not offer antispasmodics. In the event of deviations from the standard, the aim was to put in corrective measures, followed by re-exploration of compliance with patient care.

**Methods:**

This study involved a single healthcare facility with the study type being retrospective before interventions and prospective after interventions. In the first cycle, we retrieved and analysed 78 patients records whom had been suspected to have renal/ureteric colic between July and September 2022. The inclusion criteria were documented complaints of “flank/loin-to-groin pain” and diagnosis prior to performing diagnostic tests. We surveyed the first-, second-, and third-line painkillers issued, and whether an antispasmodic was given. We collected and entered data into a Microsoft Excel file and correlated it with NICE standards. Having found deviations from the standard, we conducted interventions, allowed time for clinicians to adapt, and re-explored compliance using patient case records [n=58] between February and May 2023.

**Results:**

In the 1 ^st^ cycle, 78 patients were suspected of having renal/ureteric colic. M: F = 1.2:1. Non-contrast computerized tomography of the kidney ureters and bladder (NC-CTKUB) confirmed 87% of patients with stones and 3% had no stones. NC-CTKUB was not performed in 9% of patients because they were young, and urinalysis showed no microscopic hematuria. One patient had discharged against medical advise before the NC-CTKUB was performed. Compliance with the NICE pain management guidelines for suspected renal/ureteric colic was full in 23% of cases but unsatisfactory in 78% of cases. In the 2 ^nd^ cycle, M: F = 1.5:1, NC-CTKUB was not re-audited, as the first cycle study yielded excellent results, and our action plan resulted in an NICE pain management compliance rate of 56%. Although our interventions resulted in improvements of more than twice the initial results, work still needs to be performed.

**Conclusion:**

Clinicians’ ability to correctly diagnose renal/ureteric colic is remarkable. However, the pain management compliance rate indicates room for improvement. This may be due to the limited awareness of the NICE guideline or the fact that the clinical team has an affinity for certain analgesics compared to others. We propose the need to consider select variables to existing standard guidelines to enhance compliance for improved patient care.

## Introduction

Renal/ureteric colic refers to acute and severe loin pain caused by calculi in the upper urinary tract ( Iravani et al., 2014; Weltings et al., 2021). As of 2019, the worldwide incidence was 115.55 million – a 48.57% rise over 29 years when compared to its global incidence of 77.78 million in 1990 ( Qian et al., 2022). In the United Kingdom, it is estimated that the incidence of renal/ureteric colic was 88,632 in 2019/20, which is a 2.2% increase from the 2014/15 prevalence of 86,742 ( Jour et al., 2022; Geraghty et al., 2020). This surge is likely due to lack of physical activity, dietary changes, low oral fluid intake, obesity, and global warming ( Dawson & Tomson, 2012). It is also a huge financial burden, as existing data state that in 2014/15, renal/ureteric colic gulped between £190 million and £324 million in the United Kingdom ( Geraghty et al., 2020). It is therefore reasonable to expect that, taking into consideration the effects of inflation, population increase, and rising number of persons with the disease, it is likely to cost up to £622 million by 2030 ( Tyson et al., 2019; Geraghty et al., 2020).

Although the primary function of pain stemming from renal/ureteric colic is to indicate that something is wrong, therefore, acting as a defensive and protective mechanism, the sensation is unpleasant and excruciating. When urine becomes supersaturated, it creates a favourable niche for calcium, urate, and other salts present to crystallize and form pebbles, which in turn obstructs the free flow of urine, thus causing pain ( Alelign & Petros, 2018; Papadopoulos et al., 2014). This often alters and lowers the quality of life ( Patti & Leslie, 2024). While associated pain causes anxiety ( Geraghty et al., 2020), depression ( Liu & Kelliher, 2022), sleep disorders, and immunosuppression ( Jin et al., 2023), it also results in hormonal imbalance, the most pertinent of which is initial hypercortisolemia followed by terminal hypocortisolemia ( Tennant, 2013). Increased cortisol levels can cause tachycardia, hypertension, osteoporosis, hyperlipidemia, hyperglycemia, and mental degeneration. Hypocortisolemia leads to weight loss, muscle breakdown, hypotension ( Jin et al., 2023), and sudden death ( Tennant, 2013).

As renal/ureteric colic accounts for approximately 80,000 emergency department visits annually in the United Kingdom ( Dawson & Tomson, 2012), with up to 12,000 admitted for care across the country per year ( Dawson & Dawson, 2013) and a recurrence rate of 50% at 5 – 10 years ( Alelign & Petros, 2018), and 80% at 20 years ( Patti & Leslie, 2024), it is crucial for clinicians to pay adequate attention to effective pain management as it forms an integral part of overall care given to affected patients. The aim of this study was to explore the compliance rate of the pain management pathway for patients with suspected renal/ureteric colic in a U.K. emergency department to the National Institute for Health and Care Excellence (NICE) guidelines.

## Methods

### Study design

This was a cross-sectional closed-loop clinical audit comparing current practice with NICE guidelines, which is the institute that provides evidence-based recommendations for health and care in England and Wales. The guideline states to administer a non-steroidal anti-inflammatory drug (NSAID) by any route as first-line analgesics for adults, children, and young persons with suspected renal/ureteric colic. To give intravenous paracetamol to adults, children, and young persons with suspected renal/ureteric colic if NSAIDs are contraindicated or do not provide sufficient pain relief. To consider opioids for adults, children, and young persons with suspected renal/ureteric colic if both NSAIDs and intravenous paracetamol are contraindicated or not sufficiently relieving pain. To not administer antispasmodics to adults, children, or young persons with suspected renal/ureteric colic. The standard used was that 100% of patients suspected to have renal/ureteric colic must have their pain managed according to guidelines. The study was retrospective before interventions and prospective thereafter.

### Ethics and consent

As this study was a clinical audit assessing clinicians’ adherence to NICE analgesic prescription protocol, approval with exemption of patient consent in accordance to section 60 of the Health and Social Care Act 2001, was granted by the clinical governance committee of Pilgrim Hospital, United Lincolnshire Hospitals NHS Trust. Hence, consent from patients was waived. Approval for the first cycle was granted on 13 ^th^ October, 2023, and the second cycle, on 19 ^th^ April 2023, with registration tag number P0514.

### Study setting

This study involved a single healthcare facility – Pilgrim Hospital Accidents and Emergency department. The Care Quality Commission’s Intelligent Monitoring System’s report in 2019 states that Pilgrim Hospital is a large district general, government-funded health care facility, managed by the United Lincolnshire Hospitals NHS Trust, situated in Boston, Lincolnshire, United Kingdom, providing a range of health services including round-the-clock emergency care to the communities of South and Southeast Lincolnshire ( Brown et al., 2019). It also describes its emergency department to consist of a waiting and reception area, two triage rooms, 10 major and three minor cubicles, one ‘fit to sit’ area, a ‘see and treat’ room, a plaster room, a ‘clean procedure’ room, a four-bedded resuscitation area, three rapid assessment and treatment cubicles, one waiting room and a quiet relatives room which is also used as a mental health assessment room. The report further indicated that from March 2018 to February 2019, there were over 46,000 inpatient admissions.

### Sample size and data collection

Permission was sought and granted by the Clinical Governance Department to perform a clinical audit from 18/07/2022 to 30/09/2022. A total of 260 case records of patients with flank/loin-to-groin pain, painless/painful haematuria, non-specific abdominal pain, and incidental findings of upper urinary tract stones were retrieved from the Patients Records department and reviewed. The inclusion criteria were patients presenting to the emergency department with complaints of flank/loin-to-groin pain and suspected diagnosis of renal colic prior to diagnostic tests and this was regardless of age. Patients with painless haematuria, non-specific abdominal pain, and incidental findings of calculi in the upper urinary tract were excluded. Only 78 patients met the inclusion criteria. We defined “full compliance” with NICE guideline to be patients who received NSAIDs only; however, if pain remained uncontrolled or medically unfit or allergic to NSAIDs, intravenous paracetamol was administered; however, if pain remained uncontrolled or medically unfit or allergic to paracetamol, opioids was administered – antispasmodics never given. Any deviation from this definition was regarded as “unsatisfactory compliance” with the NICE guidelines. From patient’s case notes, data was obtained using google form and then transferred into an electronic Microsoft Excel file for analysis.

### Data analysis

First, utilizing our compliance definition, we calculated the number of patients who were managed in full compliance to NICE guidelines and the number of patients who were not. Full compliance was for instance “a medically fit patient without NSAID’s allergies given per rectal diclofenac without hyoscine butyl bromide”. Unsatisfactory compliance for example was “a medically fit patient without NSAID’s allergy given intravenous paracetamol without initial trail of a NSAID” or any patient given hyoscine butyl bromide. We found full compliance only in 23% of cases. Furthermore, we checked the sensitivity of non-contrast computerized tomography of the kidney’s ureters and bladder (NC-CTKUB) in diagnosis of renal/ureteric colic in suspected patients. Lastly, we determined the male-to-female ratio.

### Qualitative study

Via a verbal discuss, the reasons given by most clinicians for unsatisfactory adherence to the NICE guideline were that they had affinity for intravenous paracetamol over NSAIDs regardless of whether patients could tolerate NSAIDs or not ( Table 1) and because they believed antispasmodics play a major role in pain management of patients with suspected renal/ureteric colic ( Figure 2).

**Table 1.** Shows the first and second cycle number of patients and the analgesics administered (n = number of patients).

	Analgesics	NSAIDs (n)	IV Paracetamol (n)	Opioids (n)	None (n)
**1** ^**st**^ **Cycle**	1 ^st^ Line	20	28	18	12
	2 ^nd^ Line	13	16	14	35
	3 ^rd^ Line	5	7	8	58
**2** ^**nd**^ **Cycle**	1 ^st^ Line	13	22	10	12
	2 ^nd^ Line	5	12	18	22
	3 ^rd^ Line	7	1	7	42

### Intervention

We developed an action plan that included apprising clinicians of the NICE guideline through an oral power-point presentation, one-on-one meetings, trust emails, and whatsapp messages. We also developed and displayed a digital poster in the emergency department and printed and pasted hard copy posters in the triage rooms.

### Post-intervention

We allowed a period of 4 months for clinicians to adapt to the national standard of pain management in patients with suspected renal/ureteric colic. We then performed a re-audit using the same inclusion and exclusion criteria, and compliance definition used in the first study. The re-audit spanned from 01/02/23 to 14/05/2023, with only 57 patients meeting the inclusion criteria having reviewed 147 patient records. Data analysis followed the same sequence used in the first cycle; however, NC-CTKUB use in the diagnosis of renal/ureteric colic was not re-analysed as the first cycle result was reassuring.

## Results

**Figure 1.**
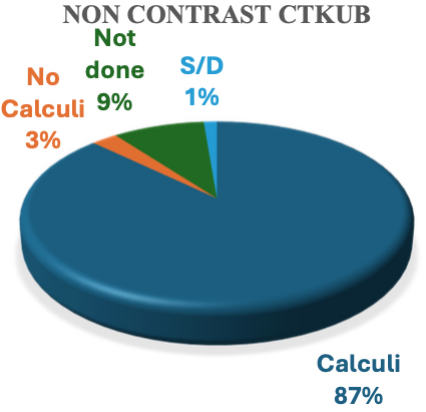
Percentage of patients suspected to have renal/ureteric colic in the first cycle study who had non-contrast computerised tomography of kidney ureter and bladder done. S/D is abbreviation for “self-discharged”.

Of the 78 patients (n = number of patients) suspected to have renal/ureteric colic in the first cycle, the male (n=43) to female (n=35) ratio was 1.2:1. NC-CTKUB confirmed that 87% (n=68) had stones and 3% (n=2) had no stones. 9% (n=7) did not have NC-CTKUB because they were young, and urinalysis showed no microscopic hematuria. 1% (n=1) self-discharged (S/D) before NC-CTKUB ( Figure 1). Overall, compliance with the NICE pain management guidelines for suspected renal/ureteric colic was full in 23% (n=18) of cases but unsatisfactory in 77% (n=60) of cases ( Figure 3). Having performed interventions, allowed a time interval of four months for clinicians to conform their practice to NICE guidelines, we performed a second cycle study - amongst the 57 patients suspected to have renal/ureteric colic, the NICE guideline was fully adhered to in 56% (n=32) but unsatisfactory in 44% (n=25) ( Figure 3). The male (n=34) to female ratio (n=23) was 1.5:1.

**Figure 2.**
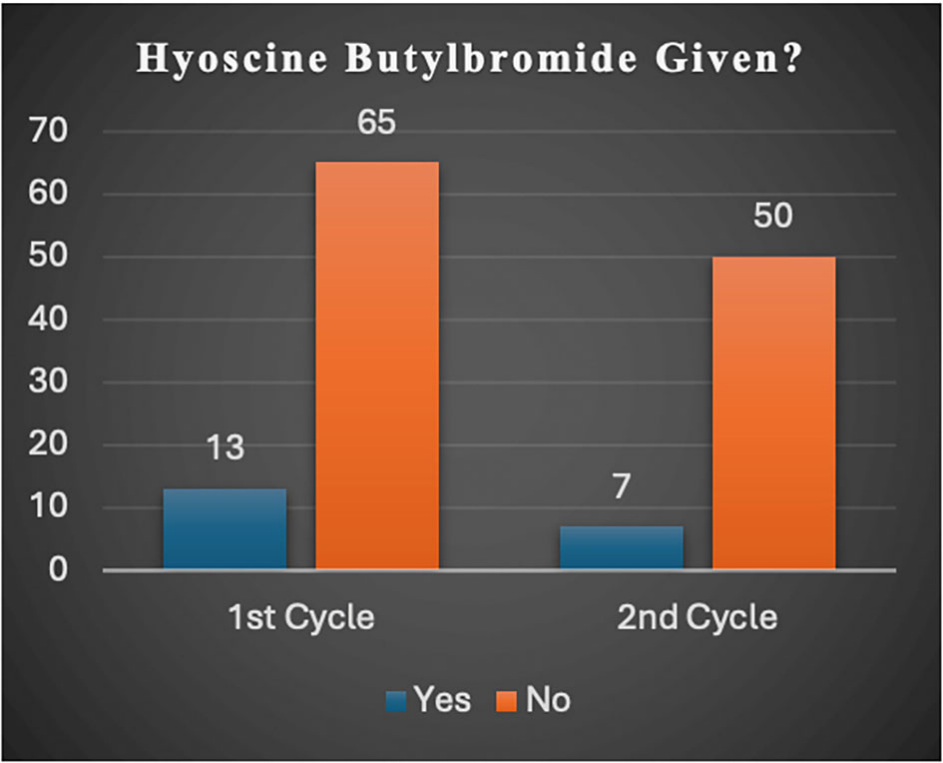
Bar chart demonstrating the number of patients given (and not given) hyoscine butyl bromide (HB) in the first and second cycle studies. HB is an antispasmodic.

**Figure 3.**
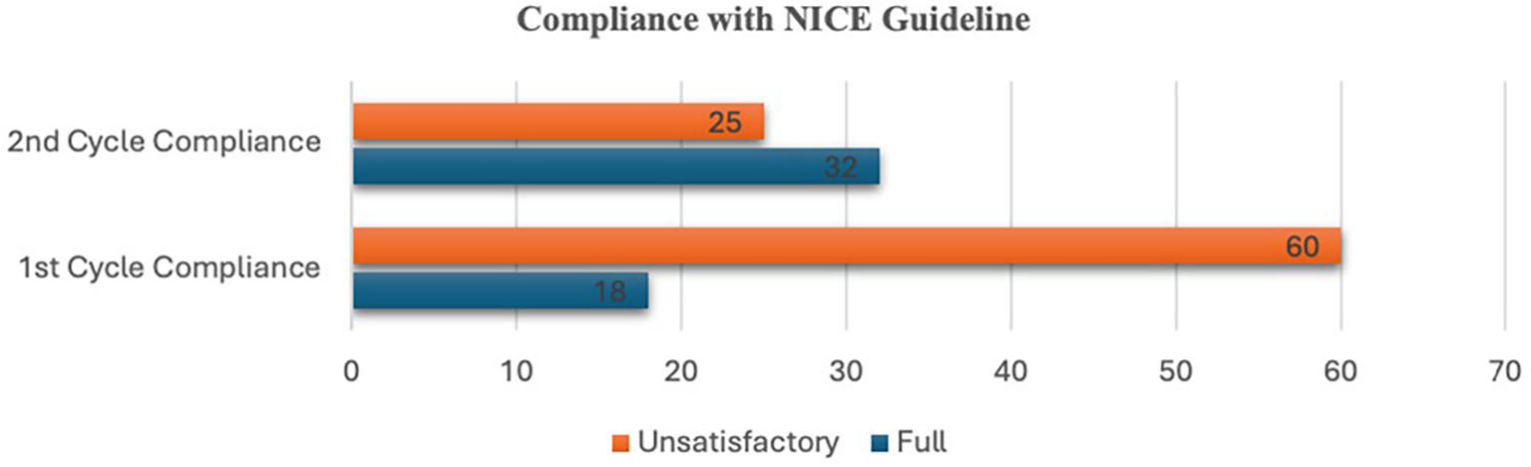
Bar chart showing compliance rate in first and second cycle studies.

## Discussion

Renal/ureteric colic has continued to remain a topical issue of discussion as its recurrence rate is high and poses a huge burden to patients as well as health care systems around the globe ( Alelign & Petros, 2018; Tyson et al., 2019). In the United Kingdom, the existing literature indicates that it is more common in males than in females ( Alelign & Petros, 2018); however, the gender gap appears to be closing ( Gillams et al., 2021). NC-CTKUB is the gold standard test ( Tyson et al., 2019), and pain management favors the administration of NSAIDs over intravenous paracetamol, opioids, and antispasmodics ( Weltings et al., 2021; NICE, 2019), as they are the most effective of the four ( Masoumi et al., 2014). Our study is consistent with its sex predisposition and diagnostic methods; however, there appears to be a disparity in the pain management pathway. We propose a need to consider the inclusion of certain variables to the existing standard guidelines.

While renal/ureteric colic has been found to be more common in males than females, the reasons remain unclear to date ( Ferraro et al., 2023). In the United Kingdom, it is stipulated that between 1990 and 2007, M:F = 2.5:1 ( Geraghty et al., 2020), which increased to 3:1 between 2016 and 2018 ( NICE, 2020) and currently, and as more females are becoming affected, the gender gap seems to be closing ( Alelign & Petros, 2018; Dawson & Tomson, 2012; Ferraro et al., 2023; NICE, 2020; Gillams et al., 2021; Feheley, 2021; Juliebo-Jones et al., 2023). For instance, a Northern Irish paper expressed it to be 1.93:1 ( Tyson et al., 2019). In our study, of the 78 patients who were suspected to have renal/ureteric colic in the first cycle, 55.1% were males and 44.9% were females corresponding to 1.2:1. In our second-cycle study involving 57 patients, 34 were males and 23 were females; therefore, corresponding to 1.5:1.

NICE (2019) advocates that all patients suspected to have renal/ureteric colic should be considered for NSAIDs, and if needed, intravenous paracetamol and opioids should be administered, and antispasmodics should not be given. Although our second-cycle results showed a compliance rate of 56%, an improvement of more than twice the initial result (23%), work still needs to be done to attain full compliance. Four reasons were found to be the cause of the deviation. First, several patients were prescribed hyoscine butylbromide: 16.7% and 13% ( Figure 2) in the first- and second-cycle studies respectively, as many clinicians, like in the Netherlands had for many years ( Weltings et al., 2021), believed that the antispasmodic effect of hyoscine butylbromide was of analgesic benefit to patients. Furthermore, many clinicians simply preferred intravenous paracetamol and oral opioids over NSAIDs, as they judged them to be safer. Most feared that the latter may cause gastrointestinal, renal, respiratory, cerebrovascular, and cardiovascular problems regardless of how medically fit the patient was. Moreso, clinicians believed that, considering the level of pain experienced by patients with suspected renal/ureteric colic, intravenous paracetamol may not be very effective; thus, it was preferable to administer it as an adjunct to oral opioids. Finally, a few clinicians simply administered analgesics, provided that it was safe and effective regardless of the standard renal/ureteric colic pain management pathway.

Arguments against and for hyoscine butylbromide have been presented. For example, although its smooth muscle relaxing effect on the ureters is yet to be demonstrated ( Weltings et al., 2021; Papadopoulos et al., 2014), continued intravenous administration is difficult ( Weltings et al., 2021) and has a rare side effect of hemodynamic instability ( Chen & Hsu, 2013), it has also been shown to have good effect on stones in the renal pelvis, less cardiac and renal side effects when compared to NSAIDs, and synergistic analgesic effects when combined with NSAIDs ( Papadopoulos et al., 2014). In support of the use of hyoscine butylbromide in the United Kingdom to manage pain associated with renal/ureteric colic, a commentary published by three nephrologists, a urologist, and a patient representative in response to NICE guidelines advises that hyoscine butylbromide should not be entirely left out of the pain management pathway but should be considered for suitable patients ( Sayer et al., 2021).

While it is true that NSAIDs are associated with Peptic Ulcer Disease (PUD), Liver and Respiratory problems, Renal failure, Myocardial and Cerebral ischemia ( Fening, 2020), they can be safely administered to select patients at the lowest effective dose and, on a short-term basis ( Bindu et al., 2020), taking into account the following factors: PUD - consider (a) per rectal diclofenac ( Patti & Leslie, 2024), (b) intramuscular ketorolac ( Patti & Leslie, 2024) (c) oral ibuprofen/aceclofenac ( Drini, 2017) administered with Proton Pump Inhibitors/H2 Receptor Antagonists/Antacids (d) celecoxib, as it has the best gastric profile ( Drini, 2017). Interestingly, concerns about the cardio- and cerebrovascular side effects of celecoxib have been described by the PRECISION study as not being different from non-selective NSAIDs ( Nissen et al., 2016). Liver Toxicity – Oral Ibuprofen and Celecoxib have been shown to have the best safety profiles ( Bindu et al., 2020). Asthma, Nasal Polyposis-Associated Chronic Rhinitis, Acute Respiratory Tract Reaction – celecoxib is not implicated; thus, it is safe ( Bindu et al., 2020). Renal failure, myocardial ischemia, and cerebral ischemia - avoid NSAID’s. Although current NSAIDs have concerning side effects, newer versions are currently in the pipeline and show promising results ( Bindu et al., 2020). These are likely to be of immense benefit to patients with suspected renal/ureteric colic.

Although the World Health Organization’s (WHO) analgesic ladder recommends paracetamol as a first-line painkiller, existing studies have proven that intravenous paracetamol is as potent ( Montazer et al., 2017) and even more effective ( Masoumi et al., 2014) than intravenous morphine in managing renal/ureteric colic. Because the side effects of opioids are worrisome, it is logical to conclude that intravenous paracetamol should be considered before opioids in affected patients.

As upper urinary tract stones can persist for prolonged periods, with affected persons experiencing agonizing pain without surgery or postoperatively ( Intermountain Healthcare, 2023), it is crucial to mention the interplay between pain and the Hypothalamic-Pituitary-Adrenal-Thyroid-Gonadal (HPATG) axis. The initial phase of severe pain causes a surge in Triiodothyronine, Thyroxine, Cortisol, Pregnenolone, DHEA, and testosterone levels, and if pain persists for a long time, serum levels fall to critical levels ( Tennant, 2013). From this, we can extrapolate the following. First, serum hormone levels can serve as biomarkers for severe and uncontrolled renal/ureteric colic. Second, it is vital to perform baseline hormonal assessments before embarking on pain management therapy, especially with opioids, as they also suppress the HPATG axis. Third, if any of the hormones is low, it is reasonable to infer that pain from renal/ureteric colic is likely severe and persisting for too long, disrupting the HPATG axis, causing associated hyperalgesia and allodynia ( Tennant, 2013). Regardless of how much analgesics is being administered, severe pain will continue until the dysregulated hormones have been attended to, thus preventing excessive and unwarranted use of analgesics. Fourth, replacement of low hormones would be lifesaving – for instance ensuring hormonal homeostasis in an individual with pain associated serum cortisol level of less than 0.1 microgram per deciliter ( Tennant, 2013) which otherwise would have been life threatening.

Of note is the clinicians’ ability to correctly diagnose renal/ureteric colic, as well as consideration for radiation risk-to-benefit. Clinicians attending to the 78 patients suspected of having renal/ureteric colic requested NC-CTKUB, and their suspicion was confirmed in 87% of cases, while 3% had no stones. NC-CTKUB was not performed in 9% of the patients because they were young, and urinalysis showed no microscopic hematuria. Its role in aiding the diagnosis of renal/ureteric colic cannot be overemphasized.

### Limitations

This study was limited given that only a second cycle study has been performed so far with an improved compliance rate to 56%. Although this is a remarkable improvement from 23%, the aim of this study remains that compliance rate should be at 100%. There is a need for future re-assessments and re-audits both locally and on a large scale.

## Conclusions

Although the literature indicates that renal/ureteric colic is significantly more common in males than in females, recent data suggest that the gap is closing, which is consistent with our study. While NICE advocates that NSAIDs should be considered before intravenous paracetamol and opioids and not to administer hyoscine butylbromide, we opine that there may be a need to accommodate hyoscine butylbromide for suitable patients and consider hormonal homeostasis in pain management workup. However, it is vital to strictly follow NICE guidelines until these suggestions are appraised. The use of NC-CTKUB in the diagnosis of renal/ureteric colic remains the gold standard, and our study demonstrated its efficiency in aiding its detection.

### Ethics and consent

As this study was a clinical audit assessing clinicians’ adherence to NICE analgesic prescription protocol, approval with exemption of patient consent in accordance with section 60 of the Health and Social Care Act 2001, was granted by the clinical governance committee of Pilgrim Hospital, United Lincolnshire Hospitals NHS Trust. Hence, consent from patients was waived. Approval for the first cycle was granted on 13 ^th^ October, 2022, and the second cycle, on 19 ^th^ April 19, 2023, with registration tag number P0514.

## Data Availability

Figshare: Retrospective study on pain management pathway for patients with suspected renal/ureteric colic in a U.K. Accidents and emergency department, https://doi.org/10.6084/m9.figshare.26810470.v1 ( Ojo et al., 2024a). This project contains the following underlying data:
• Data file 1. 1st Cycle Case Records Presenting Complaints. • Data file 2. 2nd Cycle Case Records Presenting Complaints. Data are available under the terms of the Creative Commons Zero “No rights reserved” data waiver (CC0 1.0 Public domain Dedication). The data have been thoroughly de-identified for ethical reasons. Figshare. Retrospective study on pain management pathway for patients with suspected renal/ureteric colic in a U.K. Accidents and emergency department. https://doi.org/10.6084/m9.figshare.26805784.v1 ( Ojo et al., 2024b). This project contains the following extended data:
• Audit Registration and completion forms for the 1 ^st^ and 2 ^nd^ cycle audits. • Power point presentation for the 1 ^st^ and 2 ^nd^ cycle audits. • Point presentation and letter of preliminary results. • Implementation of action plan – evidence. • Google form used for data collection in the 1 ^st^ and 2 ^nd^ cycle audits. • Approval documents for the 1 ^st^ and 2 ^nd^ cycle audits as well as implementation of action plan. Data are available under the terms of the Creative Commons Attribution 4.0 International license (CC-BY 4.0).
