# Supplementary material for: Retrospective study on pain management pathway for patients with suspected renal/ ureteric colic in a U.K. accidents and emergency department: A quality improvement project
Source: F1000Res. 2024 Sep 12;13:1052. [Version 1] doi: 10.12688/f1000research.152325.1 (PMC11937778; doi:10.12688/f1000research.152325.1)
Supplement: Peer review history

# Reviewer report: Reviewer response for version 1

**DOI:** 10.5256/f1000research.167073.r370868

**Published:** 2025-03-25

**Authors:** Samuel Davis

**Affiliations:** 1 General Surgery, Frimley Park Hospital, Frimley, England, UK

**Brief Summary**

 The article presents a retrospective quality improvement study conducted in a UK emergency department, evaluating adherence to NICE guidelines for managing suspected renal/ureteric colic. The study compares practice before and after targeted interventions aimed at improving compliance with recommended pain management pathways.

**Relevance and Significance**

 This study is important as it bridges the gap between recommended clinical practice and real-world implementation, particularly in pain management for renal colic—a common ED presentation. It also demonstrates how quality improvement initiatives can directly impact patient care and compliance with national guidelines.

**Major Points**

- The study’s design is clear, with a strong audit-reaudit methodology.
- Data supports the conclusion that guideline-based interventions improved practice.
- Authors could provide further detail on how interventions were implemented and monitored.

**Minor Points**

- Consider breaking the discussion into subheadings (e.g., "Gender Disparity", "Adherence Challenges", "Antispasmodic Use Debate") for clarity.

Is the case presented with sufficient detail to be useful for teaching or other practitioners?

Yes

Is the work clearly and accurately presented and does it cite the current literature?

Yes

If applicable, is the statistical analysis and its interpretation appropriate?

Yes

Are all the source data underlying the results available to ensure full reproducibility?

Yes

Are the conclusions drawn adequately supported by the results?

Yes

Is the background of the case’s history and progression described in sufficient detail?

Yes

Reviewer Expertise:

Urology, Surgery.

I confirm that I have read this submission and believe that I have an appropriate level of expertise to confirm that it is of an acceptable scientific standard.

# Reviewer report: Reviewer response for version 1

**DOI:** 10.5256/f1000research.167073.r334115

**Published:** 2024-10-24

**Authors:** Ayokunle Adenipekun

**Affiliations:** 1 Urology, University Hospitals Birmingham NHS Foundation Trust, Birmingham, England, UK

Good abstract. 

 Important topic as regarding pain management in A and E.   

 It is however  pertinent to state in the abstract that it is a closed cycle audit please. 

 The conclusions drawn are sensible 

 The wordings used in the methods and discussion about approval an cycles could be more succinct and also a flow cycle helps 

 There is the issue of you getting approval for second cycle before the first, but This is likely a typographical error?  

 Charts /Pies should be in 2D not 3D 

 Discussion 

 As the WHO pain ladder deviates from the NICE guideline for suspected renal /ureteric colic. This is a good point to state and explore. considering these patients are in A and E..  Hence the apparent poor compliance.   

 In addition the inclusion of the hormone pathway is not relevant and takes away from the appropriateness of this topic 

 Overall. This is a good learning article

Is the case presented with sufficient detail to be useful for teaching or other practitioners?

Yes

Is the work clearly and accurately presented and does it cite the current literature?

Yes

If applicable, is the statistical analysis and its interpretation appropriate?

Not applicable

Are all the source data underlying the results available to ensure full reproducibility?

Partly

Are the conclusions drawn adequately supported by the results?

Yes

Is the background of the case’s history and progression described in sufficient detail?

Yes

Reviewer Expertise:

Urology, clinical research

I confirm that I have read this submission and believe that I have an appropriate level of expertise to confirm that it is of an acceptable scientific standard.
